# Disgust Sensitivity and Support for Organ Donation: Time to Take Disgust Seriously

**DOI:** 10.1007/s11606-020-05734-0

**Published:** 2020-03-10

**Authors:** Lucas B. Mazur, Erik Gormsen

**Affiliations:** 1 Jagiellonian University, Krakow, Poland; 2 Sigmund Freud University, Berlin, Germany; 3 Erfurt University, Erfurt, Germany

**Keywords:** organ donation, attitudes towards organ donation, disgust, disgust sensitivity

## Abstract

**Background:**

There are currently roughly 10,000 Germans on the organ waiting list, and that number is over 113,000 in the USA. There is a clear need to increase support for organ donation in general and to increase the number of registered donors in particular.

**Objective:**

The current study examines the relationship between disgust sensitivity and attitudes towards organ donation and the possession of an organ donor card. The study also examines other important correlates of attitudes towards organ donation, such as fear, trust, and knowledge regarding organ donation.

**Design:**

The study involved an online questionnaire.

**Participants:**

Six hundred and eighteen Germans filled out an online questionnaire.

**Main Measures:**

The questionnaire contained the following measures: attitude towards organ donation, disgust sensitivity, trust towards the medical community, fear of organ donation, and knowledge regarding organ donation, as well as such demographic information as age, biological sex, degree of formal education, religious affiliation and level of religiosity, political orientation, and possession of an organ donor card.

**Key Results:**

The results replicated previous findings regarding the influence of trust and fear on attitudes towards organ donation, but only partially supported those regarding the importance of knowledge. Importantly, disgust sensitivity had a significant impact on attitudes towards organ donation, even after controlling for other variables hereto identified as important correlates in the literature (e.g., fear, trust, knowledge). What is more, there was a significant interaction between biological sex and disgust sensitivity indicating that the relationship between disgust sensitivity and attitudes towards organ donation was stronger among women than men.

**Conclusions:**

While disgust is often disregarded as a “silly,” bairnish emotion and unbefitting of discussions of serious issues such as organ donation, in line with the “affective turn” in psychology, the results of the current study suggest that in order to improve attitudes towards organ donation, we should take feelings of disgust seriously.

There are currently roughly 10,000 Germans on the organ waiting list ^1^. As of July 2019, that number is over 113,000 in the USA ^2^. There is a clear need to increase support for organ donation in general, and to increase the number of registered donors in particular. Within Germany alone, the number of donors could be more than doubled ^3^. Unless organ donation is to be somehow mandated, all approaches to increase levels of support involve a degree of personal choice, albeit to a lesser degree when opt-out laws are in place (as in the Netherlands, Austria, and Poland) than in the case of opt-in laws (as in the USA, Brazil, and Australia) ^4^. Some nations, such as Germany, have “in-between” laws which require citizens to self-identify as an organ donor or not when signing up for mandatory health insurance. As laws can help, but ultimately not solve, this challenge, it is imperative to better understand the social-psychological factors that influence levels of support for organ donation. The current research examines the relationship between attitudes towards organ donation and an emotion that is surprisingly understudied in this context, namely, disgust.

Past and present activism has placed particular emphasis on the role of education in galvanizing support for organ donation, and recent research has found support for the relationship between knowledge and attitudes towards organ donation ^5–12^. At the same time, over the last several decades, an increasing body of psychological research has pointed to the important role in shaping attitudes towards organ donation of implicit social-psychological processes, such as trust in medical professionals and trust in the medical system in general ^13 ,14^, and fear regarding organ donation ^15,11,16^, both emotional processes that need not logically follow from “what we know” ^17,18^. One particularly relevant area of research for the current study is research on disgust sensitivity ^19,20,21^. Disgust encourages humans and other animals to avoid health hazards like spoiled foods, diseased others, and other threatening substances, pathogens, and situations ^21^. It is estimated that over the course of human evolution child mortality rates before the first year, across cultures, climates and time, averaged about 27%, and that approximately 47.5% of children did not live long enough to reach puberty ^22^. Most of these deaths can be traced back to infectious diseases ^22^, making the protection against such illnesses a highly advantageous element of our evolutionary history. Surprisingly, disgust has also come to influence our moral decisions and our attitudes towards seemingly unrelated aspects of life, such as political and social issues ^17^. The question of organ donation uniquely links issues of moral decision-making, matters of life and death, and actions that for many evoke feelings of disgust, and thus constitutes an interesting and important area of research on this particular understudied emotion. While disgust is often thought of as a “silly” or “childish” emotion, and is often therefore not taken seriously within discussions of serious matters (e.g., organ donation) ^23^, it has been shown to influence consequential attitudes and important decisions to a considerable degree ^24^. The current study was therefore designed to examine the influence of disgust sensitivity on levels of support for organ donation Disgust remains surprisingly understudied within research on attitudes towards organ donation (for exceptions see ^25,26^). With the use of the measure disgust sensitivity, the current study can be considered a replication and extension of recent research wherein the primary focus was on levels of knowledge ^12^. That research ^12^ also focused solely on students of the medical profession in Germany, whereas the current study examines these variables among members of the wider German public.

## METHODS

Participants were recruited through German-speaking online forums and social media. Participation was voluntary. Participants were given the opportunity to enter a raffle to win one of three €10 Amazon vouchers. The current study consisted of an online questionnaire, containing the items listed below. Two control questions were also included to allow us to check if participants were paying attention and taking the study seriously. An incorrect answer to a control question would disqualify the data from that participant from further analysis.

### Disgust Sensitivity

To measure levels of disgust sensitivity the *Questionnaire for Assessment of Disgust Sensitivity* (*QADS*) (*Fragebogen zur Erfassung von Ekelempfindlichkeit*; *FEE*) ^27^ was used. The scale consists of 37 items, which present situations (e.g., “You smell vomit”) to be assessed on a five-point Likert-scale ranging from 1 (*not disgusting*) to 5 (*very disgusting*).

### Organ Donation Attitude Scale

The *Organ Donation Attitude Scale* (ODAS) ^28^ was adapted from the longer original measure ^29^. Participants are asked to rate eleven statements (e.g., “Organ donation leaves the body disfigured”) on a seven-point Likert-scale, ranging from 1 (*strongly disagree*) to 7 (*strongly agree*).

### Knowledge About Organ Donation

The *Knowledge-Scale*^12^ consists of 5 multiple-choice items measuring factual knowledge regarding organ donation. The items (e.g., “Which Organs cannot be donated? – Kidney/Brain/Liver/I do not know”) are of increasing difficulty.

### Fear and Trust

The *Trust-Scale* and the *Fear-Scale*^12^ each consists of three items (e.g., “I have confidence in the doctor’s decisions in the field of organ transplantation” or “I am afraid of having pain”), which participants are asked to rate on a five-point Likert-scale (1 = “I do not agree at all” to 5 = “I completely agree”). The original *Trust-Scale*^12^ consisted of five questions, two of which were excluded from the current study as they referred to specific events that are no longer widely discussed in the media and about which participants outside of the medical field are no longer likely to be aware. Following the lead of recent research ^12^, we also examined the differences between participants who already had a donor card and those who did not by means of an independent samples *t* test.

### Demographic Information

We gathered information about: age, biological sex, level of formal education, religious identification and level of religiosity, political orientation (on a scale from 1 = “left” to 5 = “right”), and whether participants were themselves recipients of an organ donation. We operationalized German identity, a contentious and widely discussed issue, as being born in Germany and having German citizenship. Not meeting this definition of German identity would disqualify the participant’s data from inclusion in the subsequent data analysis.

## RESULTS

Of the initial 680 participants, 29 were removed from further analysis as they answered at least one of the two control questions incorrectly. All participants who stated they were neither born in Germany nor had German citizenship were excluded from further analysis (*n* = 33). The final sample size was *N* = 618 (*M*_age_ = 27.0, *SD*_age_ = 9.4; 432 men, 186 women). The average level of education was 5.1 (*SD* = 1.8), which falls between 5 = “Ausbildung” (vocational training) and 6 = “Bachelor”. The majority of participants identified as belonging to no religion (55.6%), followed by Christians (44.4%). Regarding religiosity (*M* = 0.7, *SD* = 1.0), 60.7% reported that they were not religious at all, 18% that they were a bit religious, 12.2% that they were somewhat religious, 6.3% that they were relatively religious, and 2.4% (*n* = 15) that they were very religious. Out of 584 participants who filled out the one-item scale of political orientation (*M* = 2.3, *SD* = 0.9), 17.2% described themselves as very left leaning, 43.5% as relatively left leaning, 30.3% as politically neutral, 8.6% as relatively right leaning, and 0.3% as very right leaning. Two participants had themselves received an organ.

All of the scales showed good internal consistency: QADS/FEE *α* = .89, *M* = 2.7, *SD* = 0.5; ODAS *α* = .83, *M* = 5.7, *SD* = 1.0; Trust *α* = .67, *M* = 5.7, *SD* = 1.0; Fear *α* = .79, *M* = 1.2, *SD* = 0.7 (which increased from *α* = .67 after removing item 3). The mean score on the Knowledge-Scale was 2.44 (*SD* = 0.9).

Bivariate correlations indicate that attitudes towards organ donation significantly correlated at the *p* < .05 level with disgust sensitivity (*r* = − .09), trust (*r* = .50), fear (*r* = − .42), level of formal education (*r* = − .17), religiosity (*r* = − .18), political orientation (*r* = − .26). There was no correlation with knowledge about organ donation (*r* = .053). Similar to previous findings ^12^, participants who already had a donor card were more knowledgeable about organ donation, although their level of formal education did not significantly differ. Participants with a donor card also reported lower levels of fear, higher levels of trust, and lower levels of disgust sensitivity than participants who did not have a donor card (see Table 1).

**Table 1:** Results of *t* Test and Descriptive Statistics for Selected Variables by Ownership of Donor Card

Variable	Donor card ownership						95% CI for mean difference			
	No			Yes						
	*M*	*SD*	*n*	*M*	*SD*	*n*		*t*	*d*	*p*
Organ donation attitude	5.09	1.12	203	6.02	0.71	415	0.78, 1.07	**12.48**	**1.074**	**.000**
Disgust sensitivity	3.00	0.55	203	2.85	0.48	415	− 0.24, − 0.06	− **3.44**	− **0.298**	**.001**
Knowledge	2.26	1.13	203	3.00	1.02	415	0.57, 0.92	**8.19**	**0.700**	**.000**
Trust	3.42	0.97	203	4.01	0.76	415	0.45, 0.73	**8.26**	**0.707**	**.000**
Fear	2.22	0.83	203	1.50	0.81	415	− 0.08, − 0.88	− **8.67**	− **0.882**	**.000**
Age	28.23	12.30	203	26.34	7.49	415	− 3.46, − 0.32	− **2.36**	− **0.202**	**.019**
Education	5.02	1.46	202	5.08	1.28	415	− 0.17, 0.28	0.48	0.045	.634
Religiosity	0.85	1.11	203	0.59	0.95	415	− 0.43, − 0.09	− **3.03**	− **0.259**	**.003**
Political orientation	2.51	0.85	190	2.20	0.85	394	− 0.46, − 0.17	− **4.19**	− **0.365**	**.000**

Men and women were equally likely to possess a donor card (*χ*^2^ = 1.101, *p* = 0.29). There were no differences between men and women when it came to attitudes towards organ donation (*p* = .96), knowledge about organ donation (*p* = .33), or levels of formal education (*p* = .38). There were, however, significant differences between men and women in levels of disgust sensitivity, trust, and fear (see Table 2).

**Table 2:** Differences Between Men and Women on Selected Measures

	Men			Women			
	*M*	*SD*		*M*	*SD*	*t*	*p*
Disgust sensitivity	5.09	1.12		6.02	0.71	**12.48**	**.000**
Trust	0.85	1.11		0.59	0.95	− **3.03**	**.003**
Fear	2.51	0.85		2.20	0.85	− **4.19**	**.000**

A multiple regression run with mean-centered variables yielded a significant model, *F*(10, 573) = 30.210, *p* < .00, *R*^2^ = .345 (see Table 3). Importantly, disgust sensitivity was a significant predictor in the model. While indicating that factors outside the model are also influencing attitudes towards organ donation, the percentage of explained variance is not without significance within social and psychological research where causal relations are more distally connected than normally seen in the biological sciences. Given the differences between male and female participants on several key variables, and in light of biological sex differences in general levels of disgust sensitivity reported in the literature ^30,31^, we included the interaction term between biological sex and disgust sensitivity. The interaction term was also significant, which in this case means that the negative relationship between disgust sensitivity and support for organ donation was stronger for women than for men. In other words, disgust sensitivity significantly predicted attitudes to organ donation, and this relationship was stronger for women than for men. What is even more interesting about these findings is that the measure of disgust sensitivity assesses a general tendency to find a wide range of situations disgusting, none of which are directly related to organ donation. As the measures of fear and trust were directly related to organ donation (e.g., fear of organ donation), rather than measuring general levels of global fearfulness or trust, it is not surprising that they would be stronger predictors of organ donation attitudes. That a general measure of disgust sensitivity is predictive of such attitudes suggests just how important this emotion is. Interestingly, contrary to past research ^12^, neither knowledge about organ donation nor level of formal education were significant predictors of support for organ donation.

**Table 3:** Multiple Regression Analysis for Selected Variables by Organ Donation Attitude

	*B*	*SE B*	*β*	*t*	*p*
Disgust sensitivity	.493	.201	.260	2.453	.014
Knowledge	− .012	.030	− .014	− .419	.676
Trust	.354	.043	.320	8.287	.000
Fear	− .254	.036	− .273	− 7.032	.000
Sex	.265	.080	.123	3.324	.001
Age	− .008	.004	− .073	− 1.930	.054
Education	− .044	.026	− .061	− 1.670	.095
Religiosity	− .067	.034	− .070	− 1.979	.048
Political orientation	− .147	.040	− .132	− 3.717	.000
Disgust sensitivity*sex	.387	.141	− .295	− 2.746	.006

## DISCUSSION

The current study provides support for the significant role of disgust sensitivity – a general sensitivity to this emotion – in reducing support for organ donation. The more participants were prone to feel disgust, the less supportive they were of organ donation. Not only did participants with a donor card show lower levels of generalized disgust than participants without a donor card, but disgust sensitivity remained a significant predictor of attitudes towards organ donation after controlling for other variables shown in the literature to be important, such as fear, trust, and knowledge. The effect of disgust sensitivity on organ donation attitudes was stronger among women than among men. Fear and trust also significantly predicted levels of support for organ donation, and levels of both emotions differed as expected among participants with and without a donor card. While knowledge about organ donation differed between these two groups, it was no longer a significant predictor of organ donation attitudes after controlling for the other variables under consideration.

There are several important limitations of the current study, beyond its national (German) context. For example, political orientation and religiosity need to be interpreted with caution as our sample was overwhelmingly and disproportionately liberal and non-religious. It would be helpful to recruit participants with a wider range of political and religious beliefs. The sample is also not representative with regard to biological sex or age. Future research should also consider whether participants have had close contact with donors or organ recipients in their personal life, and whether they or close relations have experienced major medical issues when a transplant organ was needed. Future research should also include additional measures of disgust that are directly linked to organ donation, rather than the general sensitivity to this emotion as examined here. With such measures, we assume that the relationship between disgust and attitudes to organ donation will be even stronger.

These limitations aside, the current study suggests that disgust sensitivity, along with fear and trust, does in fact significantly impact levels of support for organ donation, and that there may be meaningful differences in this regard between men and women. While not disregarding the importance of knowledge, we hope the current findings encourage the medical community and organ donation advocates to pay more attention to the findings of the “affective turn” in psychology ^18^, whereby implicit emotions are understood to play a significant role in shaping our attitudes and decision-making. Disgust remains an underappreciated and frequently disregarded emotion, and it is often deemed inappropriate and childish in various situations ^23^. We should support education efforts, but also work to change how people emotionally respond to such deeply personal matters as organ donation. While we increasingly appreciate the influence of fear and trust when it comes to attitudes towards organ donation, and campaigns have been designed to address these emotions, we should also develop strategies to address the very serious matter of disgust.

## References

[1] Deutsche Stiftung Organtransplantation (2018). Retrieved from: https://www.dso.de/uploads/tx_dsodl/JB_2017_web_01.pdf&sa=D&ust=1537358425859000&usg=AFQjCNEJ4Aaw87DTxl5dy9y_AE8OzOvkag

[2] United Network for Organ Sharing (nd). Statistics accessed via unos.org

[3] Wesslau C, Grosse K, Krüger R (2006). Transplationsmedizin: Organspender-Potenzial ist nicht ausgeschöpft. Deutsches Arztblatt.

[4] Cohen C (1992). The case for presumed consent to transplant human organs after death. Transplantation Proceedings..

[5] Bharambe VK, Rathod H, Angadi K. Knowledge and attitude regarding organ donation among medical students. *BANTO Journal.* 2016; 14(1): 34–40.

[6] Figueroa CA, Mesfum ET, Acton NT, Kunst AE. Medical students’ knowledge and attitudes towards organ donation: Results of a Dutch survey. *Transplantation Proceedings*. 2013; 45: 2093–2097. 10.1016/j.transproceed.2013.02.135 23953518

[7] Sander SL, Miller BK. Public knowledge and attitudes regarding organ and tissue donation: an analysis of the northwest Ohio community. *Patient Education and Counseling*, 2006; 58: 154–163. 10.1016/j.pec.2004.08.003 16009291

[8] Saub EJ, Shapiro J, Radecki S (1998). Do patients want to talk to their physicians about organ donation? Attitudes and knowledge about organ donation: A study of Orange County, California residents. Journal of Community Health.

[9] Schaeffner ES, Windisch W, Freidel K, Breitenfeldt K, Winkelmayer WC (2004). Knowledge and attitude regarding organ donation among medical students and physicians. Transplantation..

[10] Shanteau J. Psychological and behavioral factors in organ donation. *Organ Donation and Transplantation – an Interdisciplinary Approach.* 2013; 91–104.

[11] Sebastian-Ruiz MJ, Guerra-Saenz EK, Vargas-Yamanaka AK, et al. Knowledge and attitude towards organ donation on medicine students of a Northeastern Mexico Public University. *Gaceta Médica de México.* 2017; 153: 395–404. 10.24875/GMM.17002573 28991282

[12] Terbonssen T, Settmacher U, Wurst C, Dirsch O, Dahmen, U. Attitude towards organ donation in German medical students. *Langenbecks Archives of Surgery.* 2016; 401: 1231–1239. 10.1007/s00423-016-1482-4 27443849

[13] Khoddami-Vishteh HR, Ghorbani F, Ghasemi AM, Shafaghi S, Najafizadeh K. Attitude towards organ donation: A survey on Iranian teachers. *Transplantation Proceedings.* 2011; 43(2): 407–409. 10.1016/j.transproceed.2011.01.052 21440718

[14] Teixeira RK, Goncalves TB, Silva JA. Is the intention to donate organs influenced by the public’s understanding of brain death*? The Revista Brasileira de Terapia Intensiva.* 2012; 24(3): 258–262. 23917827

[15] Demir T, Selimen D, Yildirim M, Kucuk HF. Knowledge and attitudes towards organ/tissue donation and transplantation among health care professionals working in organ transplantation or dialysis units. *Transplantation Proceedings.* 2011; 43:1425–1428. 10.1016/j.transproceed.2011.01.167 21693211

[16] Uhlig CE, Koch R, Promesberger J, et al. Attitudes towards postmortem cornea donation in Germany: A multicenter survey. *Graefe’s Archive for Clinical and Experimental Ophthalmology.* 2014; 252(12): 1955–1962. 10.1007/s00417-014-2796-y 25236759

[17] Horberg EJ, Oveis C, Keltner D, Cohen AB. Disgust and the moralization of purity. *Journal of Personality and Social Psychology.* 2009; 97(6): 963–976. 10.1037/a0017423 19968413

[18] May J. Does disgust influence moral judgment. *Australasian Journal of Philosophy.* 2014; 92(1): 125–141.

[19] Shook NJ, Oosterhoff B, Terrizzi Jr. JA, Brady KM. “Dirty politics”: The role of disgust sensitivity in voting. *Translational Issues in Psychological Science.* 2017; 3(3): 284–297.

[20] Van Leeuwen F, Dukes A, Tybur JM, Park JH. Disgust sensitivity relates to moral foundations independent of political ideology. *Evolutionary Behavioral Sciences.* 2017; 11(1): 92–98.

[21] Inbar Y, Pizarro D, Iyer R, Haidt J. Disgust sensitivity, political conservatism, and voting. *Social Psychological and Personality Science.* 2011; 3(5): 537–544.

[22] Volt AA, Atkinson JA. Infant and child death in the human environment of evolutionary adaptation. *Evolution and Human Behavior.* 2013; 34(3): 182–192.

[23] Strohminger N. Disgust talked about. *Philosophy Compass.* 2014; 9(7): 478–493.

[24] Sherman NC, Sherman MF, Smith RJ, Rickert-Wilbur P. Disgust sensitivity and attitudes towards organ donation among African-American college students. *Psychological Reports.* 2001; 89: 11–23. 10.2466/pr0.2001.89.1.11 11729529

[25] O’Carroll RE, Foster C, McGeechan G, Sanford K. The “ick” factor, anticipated regret, and willingness to become an organ donor. *Health Psychology.* 2011; 30: 236–245. 10.1037/a0022379 21401258

[26] Morgan SE, Stephenson MT, Harrison TR, Afifi WA, Long SD (2008). Facts versus “feelings”: How rational is the decision to become an organ donor?. Journal of Health Psychology..

[27] Schienle A, Walter B, Stark J, Vaitl D. Ein Fragebogen zur Erfassung der Ekelempfindlichkeit (FEE). *Zeitschrift für Klinische Psychologie und Psychotherapie: Forschung und Praxis.* 2002; 31: 110–120.

[28] Sharpe E, Moloney G, Sutherland M, Judd A (2017). The power of an immediate donor registration opportunity: Translating organ donation attitudes into registration behavior. Basic and Applied Social Psychology..

[29] Parisi N, Katz I. Attitudes towards posthumous organ donation and commitment to donate. *Health Psychology.* 1986; 5(6): 565–580. 10.1037//0278-6133.5.6.565 3803350

[30] Bassett JF (2017). Disgust sensitivity accounts for some but not all gender differences in death attitudes. OMEGA-Journal of Death and Dying..

[31] Olatunji BO, Sawchuk CN, Arrindell WA, Lohr JM (2004). Disgust sensitivity as a mediator of the sex differences in contamination fears. Personality and Individual Differences..

